# RUNX1 promotes denervation-induced muscle atrophy by activating the JUNB/NF-κB pathway and driving M1 macrophage polarization

**DOI:** 10.1515/biol-2025-1157

**Published:** 2025-08-18

**Authors:** Wei Hu, Yang Huang, Wei Yin, Yao Huang, Jian Wu

**Affiliations:** Department of Radiology, Xianning Central Hospital, The First Affiliated Hospital of Hubei University of Science and Technology, No. 228, Jingui Road, Xian’an District, Xianning, Hubei, 437000, China

**Keywords:** RUNX1, JUNB, NF-κB signaling, muscle atrophy, macrophage

## Abstract

Peripheral nerve injury-induced muscle atrophy is characterized by chronic inflammation and dysregulated macrophage polarization. RUNX1, a transcription factor upregulated in denervated muscle, has been implicated in linking muscle degeneration to inflammatory processes, but its downstream targets and mechanisms remain unclear. The aim of this study is to delineate the RUNX1-JUNB-NF-κB axis in driving inflammation-mediated muscle atrophy. The GSE183802 single-nucleus RNA sequencing dataset was analyzed to identify RUNX1-associated pathways. A sciatic nerve transection model in mice was established to validate RUNX1 expression dynamics. Chromatin immunoprecipitation, dual-luciferase reporter assays, and siRNA-mediated knockdown were used to confirm RUNX1’s transcriptional regulation of *JUNB*. *In vitro* models (C2C12 myotubes, RAW 264.7 macrophages) assessed RUNX1-driven inflammatory responses, NF-κB activation, and extracellular matrix remodeling. RUNX1 was significantly upregulated in denervated muscle, particularly in myonuclei and macrophage subclusters, correlating with elevated atrophy markers (MuRF1, Atrogin-1). RUNX1 overexpression directly activated *JUNB* transcription via promoter binding, leading to NF-κB pathway activation (increased p65 phosphorylation) and M1 macrophage polarization (enhanced IL-1β/TNF-α secretion). JUNB knockdown reversed RUNX1-induced pro-inflammatory cytokine release, NF-κB signaling, and muscle atrophy markers. This study identifies the RUNX1-JUNB-NF-κB axis as a central regulator of inflammation-driven muscle atrophy following denervation. Targeting this pathway may offer therapeutic potential to mitigate neurogenic muscle degeneration and immune-mediated damage in conditions such as peripheral nerve injuries or motor neuron diseases.

## Graphical abstract

**Figure:**
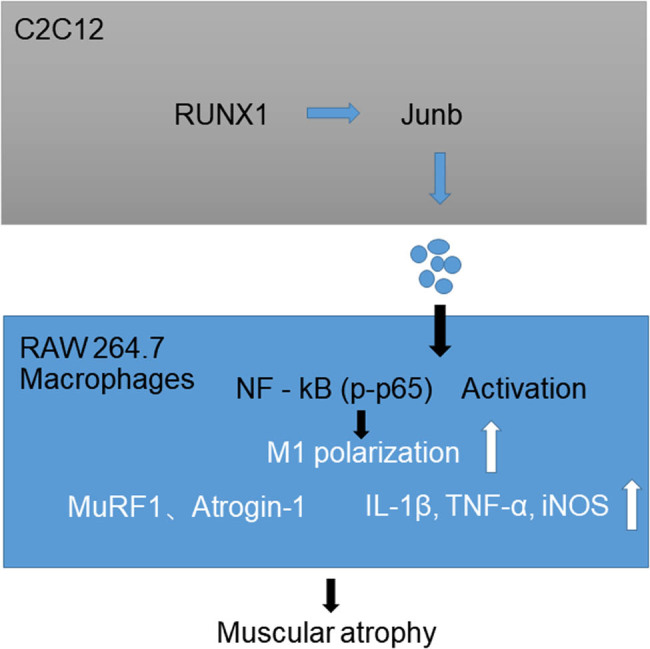

## 1 Introduction

Skeletal muscle atrophy, characterized by a reduction in muscle mass and function, is a debilitating condition associated with chronic diseases, including neuromuscular disorders, cancer, and cachexia [1]. This condition severely impacts patients’ quality of life and contributes to increased healthcare costs [2]. Peripheral nerve injuries (PNIs), a leading cause of muscle atrophy, result in neuromuscular junction (NMJ) denervation and impaired muscle function. Macrophages are critically involved in NMJ reinnervation through debris clearance, modulation of inflammation, and support for axonal regeneration. Dysregulated macrophage activity may hinder NMJ recovery and worsen muscle atrophy. For instance, in Schwann cell-specific *Gpr126* mutants, reduced macrophage infiltration and imbalanced chemokine expression (e.g., elevated Ccl2 and reduced Tnfα) delay NMJ reinnervation, highlighting the importance of coordinated macrophage responses for effective muscle recovery [3].

Recent research has identified RUNX1 as a key transcription factor in skeletal muscle denervation and atrophy, serving as an early marker of NMJ dysfunction and muscle stress [4]. In aging mouse models, RUNX1 upregulation correlates with NMJ instability preceding sarcopenia and regulates denervation-related genes, including acetylcholine receptor subunits and atrophy markers such as *MuRF1* and *Atrogin-1* [5,6]. While RUNX1 initially supports adaptive responses, its sustained expression exacerbates delayed recovery and muscle susceptibility to atrophy. Notably, RUNX1 also modulates macrophage polarization and immune signaling, particularly in denervation-prone muscles like the tibialis anterior, with expression patterns influenced by sex and exercise [7].

The JUNB-NF-κB axis, a well-characterized inflammatory pathway, plays a pivotal role in muscle pathology. JUNB, an AP-1 family transcription factor, regulates pro-inflammatory cytokine production (e.g., IL-1β, TNF-α) and interacts with NF-κB to amplify inflammatory responses [8]. NF-κB, a master regulator of inflammation, drives muscle atrophy by inducing proteolytic systems (e.g., ubiquitin-proteasome) and suppressing anabolic pathways [9]. While previous studies have investigated RUNX1 in contexts such as fibrosis and inflammation [7,8,9], and JUNB-NF-κB crosstalk has been implicated in chronic inflammatory diseases, the direct regulatory relationship between RUNX1, JUNB, and NF-κB in denervation-induced muscle atrophy remains underexplored. This study provides the first evidence that RUNX1 binds directly to the JUNB promoter, activating the JUNB-NF-κB pathway to mediate macrophage polarization and subsequent muscle degeneration following denervation.

Building on this foundation, we hypothesized that RUNX1 exacerbates denervation-induced atrophy by activating the JUNB-NF-κB axis, thereby amplifying inflammation and extracellular matrix (ECM) dysregulation. Leveraging the GSE183802 single-nucleus RNA sequencing (snRNA-seq) dataset, we identified RUNX1 upregulation in myonuclei and macrophage subclusters post-denervation. Using a sciatic nerve transection model, we demonstrated that RUNX1 drives macrophage M1 polarization and muscle atrophy markers. Mechanistically, RUNX1 directly binds to the *JUNB* promoter, activating NF-κB signaling (via p65 phosphorylation) and perpetuating a pro-inflammatory cascade. These findings establish the RUNX1-JUNB-NF-κB axis as a central driver of neurogenic muscle degeneration, offering novel therapeutic targets for mitigating atrophy in PNIs and related disorders.

## 2 Methods

### 2.1 snRNA-seq analysis

To explore gene expression changes associated with denervation-induced muscle atrophy, the GSE183802 snRNA-seq dataset of gastrocnemius (GAS) muscles from normal and denervated mice was obtained, and after quality control, 29,539 nuclei were retained (15,739 normal; 13,800 denervated). Using Seurat’s FindClusters (tested resolutions 0.2–1.0; final resolution = 0.5), we partitioned cells into 12 populations including different myofiber subtypes (Type I, Type IIa, IIx, IIb), fibro-adipogenic progenitors (FAPs), muscle stem cells (MuSCs), macrophages, and other supporting cell types.

### 2.2 Sciatic nerve transection model

A total of six female Sprague-Dawley rats (180–220 g) were randomly divided into two groups: Group A (Sham surgery, *n* = 3) and Group B (Denervation model, *n* = 3). Group A underwent a sham operation, where an incision was made to expose the left tibial nerve without transection, followed by wound closure with 3-0 silk sutures. In Group B, the left tibial nerve was completely transected, and the proximal stump was ligated with 5-0 nylon sutures to prevent reinnervation. All surgical procedures were performed under sterile conditions by two experienced microsurgeons. Anesthesia was induced with 3% sodium pentobarbital (50 mg/kg, intraperitoneally). A longitudinal incision was made over the left hindlimb, and the tibial nerve was carefully isolated under a surgical microscope. The nerve was completely transected at mid-thigh level, and the proximal stump was tightly ligated to ensure no reconnection. Postoperatively, all rats were placed in clean cages with fresh bedding and monitored for signs of distress or infection. Four weeks after surgery, rats were euthanized, and the GAS muscle from both groups was carefully dissected. The collected muscle tissues were divided into two parts: one was fixed in 4% paraformaldehyde for hematoxylin and eosin (HE) staining to assess muscle morphology and atrophy, while the other was stored at −80°C for subsequent RNA extraction and quantitative real-time PCR (qRT-PCR) analysis to evaluate the expression of atrophy-related genes.


**Ethical approval:** The research related to animal use has been complied with all the relevant national regulations and institutional policies for the care and use of animals, and has been approved by the Animal Research Ethics Committee of Xianning Central Hospital (The First Affiliated Hospital of Hubei University of Science and Technology) (Approval No. 2023-97).

### 2.3 Histology and morphometric analysis

The GAS muscles were harvested from both the experimental and control groups. The muscles were dissected, weighed, and immediately frozen in liquid nitrogen-cooled isopentane. Next 10 μm thick transverse cryosections were prepared using a cryostat and stained with HE dye (Thermo Fisher Scientific, Rockford, IL, USA) following standard protocols. The stained sections were examined and imaged using an Eclipse TE 2000-U microscope equipped with a Digital Sight DS-Fi1 camera (Nikon, Tokyo, Japan). Muscle fiber morphometry was analyzed in a blinded manner using ImageJ software (NIH, Bethesda, MD, USA). Approximately 500 myofibers were measured in each of five non-adjacent transverse sections per muscle (totaling ∼2,500 fibers per rat) to determine cross-sectional area (CSA) and minimal Feret’s diameter. These parameters were used to quantitatively assess muscle atrophy and morphological changes following sciatic nerve transection.

### 2.4 C2C12 myotube and macrophage co-culture model

RAW 264.7 (CL-0190) and C2C12 (CL-0044) were obtained from ATCC. The cells were cultured in Dulbecco’s Modified Eagle’s Medium (DMEM, Gibco, United States) supplemented with 10% fetal bovine serum (Gibco, United States) under a humidified atmosphere with 5% CO_2_ at 37°C. The cell lines were authenticated using short tandem repeat analysis performed in June 2024 at the Laboratory Animal Center of Wuhan University. All experiments were conducted with mycoplasma-free cells, confirmed by the absence of detectable mycoplasma signals in Hoechst 33342-stained cells (Supplementary file). A 6-well and 24-well transwell co-culture system (Corning, United States) was used for the non-contact co-culture of RAW264.7 macrophages and C2C12 myoblasts. For C2C12 overexpression, C2C12 myoblasts were transfected with either an empty vector (NC) or a RUNX1 overexpression plasmid (oe-RUNX1) using Lipofectamine 3000 (Thermo Fisher Scientific, United States) according to the manufacturer’s instructions. Cells were incubated with the transfection mixture for 6 h, after which the medium was replaced with fresh DMEM supplemented with 10% fetal bovine serum. 24 h post-transfection, successful overexpression was confirmed via qRT-PCR and Western blot analysis.

For co-culture, 2 × 10^6^ and 4 × 10^5^ RAW264.7 macrophages were plated in the upper chamber of 6-well and 24-well transwell plates, respectively. Meanwhile, 6.6 × 10^6^ and 13.3 × 10^5^ C2C12 myoblasts (NC or oe-RUNX1-transfected) were plated in the lower chamber of the respective transwell plates. The macrophage-to-myoblast ratio was maintained at approximately 30%, reflecting physiological conditions in skeletal muscle. Cells were allowed to adhere for 24 h before initiating co-culture. To induce M1 polarization, RAW264.7 macrophages were treated with lipopolysaccharide (LPS) (100 ng/mL) and interferon-gamma (IFN-γ) (50 ng/mL) for 24 h before co-culture. The co-culture was maintained for 5 days in DMEM supplemented with 2% horse serum (Gibco, United States) to promote C2C12 myogenic differentiation. After co-culture, macrophages and myotubes were separately collected for further biochemical and molecular analyses, and culture supernatants were collected for cytokine assays.

### 2.5 Total RNA isolation and qRT-PCR

RNA was extracted from cells and GAS using Trizol reagent (Qiagen, Germany). Complementary DNA (cDNA) was synthesized according to the manufacturer’s instructions of the SensiFAST™ cDNA Synthesis Kit (Bioline, OH, USA). Real-time PCR was performed with Power SYBR™ Green PCR Master Mix (Applied Biosystems, MA, USA). The cycling protocol was: initial denaturation at 95°C for 2 min; 40 cycles of 95°C for 15 s and 60°C for 30 s; followed by melt-curve analysis from 60 to 95°C. GAPDH served as the endogenous control, and relative expression was calculated by the 2^–ΔΔCt^ method. All reactions were run in technical triplicate and biological triplicates. The primers used for real-time PCR are listed in Table 1.

**Table 1:** Primer sequences

Genes	Primers	
	Forward (5′−3′)	Reverse (5′−3′)
RUNX1 (Rattus norvegicus)	TACCTGGGGTCCATCACCTC	GGTCCGGGGCTGTTGAGA
RUNX1 (Mus musculus)	CCTTCAGGAGAGGTGCGTTT	CTCGTGCTGGCATCTCTCAT
JUNB (Mus musculus)	ATCAGACACAGGCGCATCTC	TGACAAAACCGTCCGCAAAG
MuRF1 (Mus musculus)	CAGAGGGTAAAGAAGAACACCA	TGCTAGTCCCTGCTCTCTGA
Atrogin-1 (Mus musculus)	TGAGCGACCTCAGCAGTTAC	GCGCTCCTTCGTACTTCCTT
GAPDH (Rattus norvegicus)	CTCAGTTGCTGAGGAGTCCC	ATTCGAGAGAAGGGAGGGCT
GAPDH (Mus musculus)	TGGAGCCAAAAGGGTCATCA	ATGAGCCCTTCCACAATGCC

### 2.6 Western blot analysis

Total protein was extracted from cells and GAS tissues, and protein concentrations were determined using the BCA protein assay kit. The proteins were separated by SDS-PAGE and transferred onto polyvinylidene fluoride membranes. The membranes were blocked with 5% nonfat milk for 1 h at room temperature to prevent non-specific binding. After blocking, the membranes were incubated overnight at 4°C with primary antibodies against RUNX1 (ab240639, 1:500, Abcam), JUNB (ab168356, 1:500, Abcam), CD86 (ab239075, 1:500, Abcam), NF-kB p65 (ab32536, 1:500, Abcam), and β-actin (ab5694, 1:1,000). The following day, the membranes were washed with tris-buffered saline with Tween 20 and incubated with horseradish peroxidase-conjugated secondary antibodies for 1 h at room temperature. The protein bands were visualized using enhanced chemiluminescence reagent (Amersham Biosciences, UK) and detected with a ChemiDoc MP system (Bio-Rad, USA). Band intensity was quantified using ImageJ software, and the protein expression was normalized to β-actin.

### 2.7 Enzyme-linked immunosorbent assay (ELISA)

ELISA was used to measure the levels of inducible nitric oxide synthase (iNOS), tumor necrosis factor-alpha (TNF-α), and interleukin-1 beta (IL-1β*) in the supernatants of RAW264.7 macrophages. Cytokine concentrations were determined using commercial ELISA kits (Absin Bioscience, China) according to the manufacturer’s instructions. All samples were analyzed in duplicates.

### 2.8 Chromatin immunoprecipitation (ChIP) assay

ChIP assay was performed to analyze the binding of RUNX1 to the promoter regions of JUNB in C2C12 myoblasts. Briefly, cells were cross-linked with 1% formaldehyde for 10 min at room temperature and quenched with 0.125 M glycine. The chromatin was then sheared using sonication to obtain fragments of 200–500 bp. Immunoprecipitation was carried out using specific antibodies against RUNX1, with IgG as a negative control. The immunoprecipitated DNA was eluted, purified, and analyzed by qRT-PCR to quantify the binding of RUNX1 to the promoter regions of interest.

### 2.9 Luciferase reporter assay

The luciferase reporter assay was performed using the Dual-Luciferase Reporter Assay Kit (Promega). HEK293T cells were cotransfected with wild-type (WT) or mutant (MUT) JUNB promoter plasmids and the RUNX1 overexpression plasmid. Mutations were introduced in the predicted binding sites 2 and 3 of the JUNB promoter. Negative control plasmids were included. After 48 h, luciferase activities were measured, with Firefly luciferase used to assess RUNX1 binding to the JUNB promoter and Renilla luciferase for normalization. Data were quantified using a luminometer (Promega).

### 2.10 Statistical analysis

All data are presented as mean value ± standard deviation. Statistical analyses were performed using GraphPad Prism (version 9.0). Differences between two groups were assessed using the unpaired Student’s *t*-test. Multiple-group comparisons were conducted using one-way ANOVA followed by Tukey’s post-hoc test. A *P*-value <0.05 was considered statistically significant.

## 3 Results

### 3.1 snRNA-seq reveals RUNX1 upregulation in denervation-induced muscle atrophy

**Figure 1:**
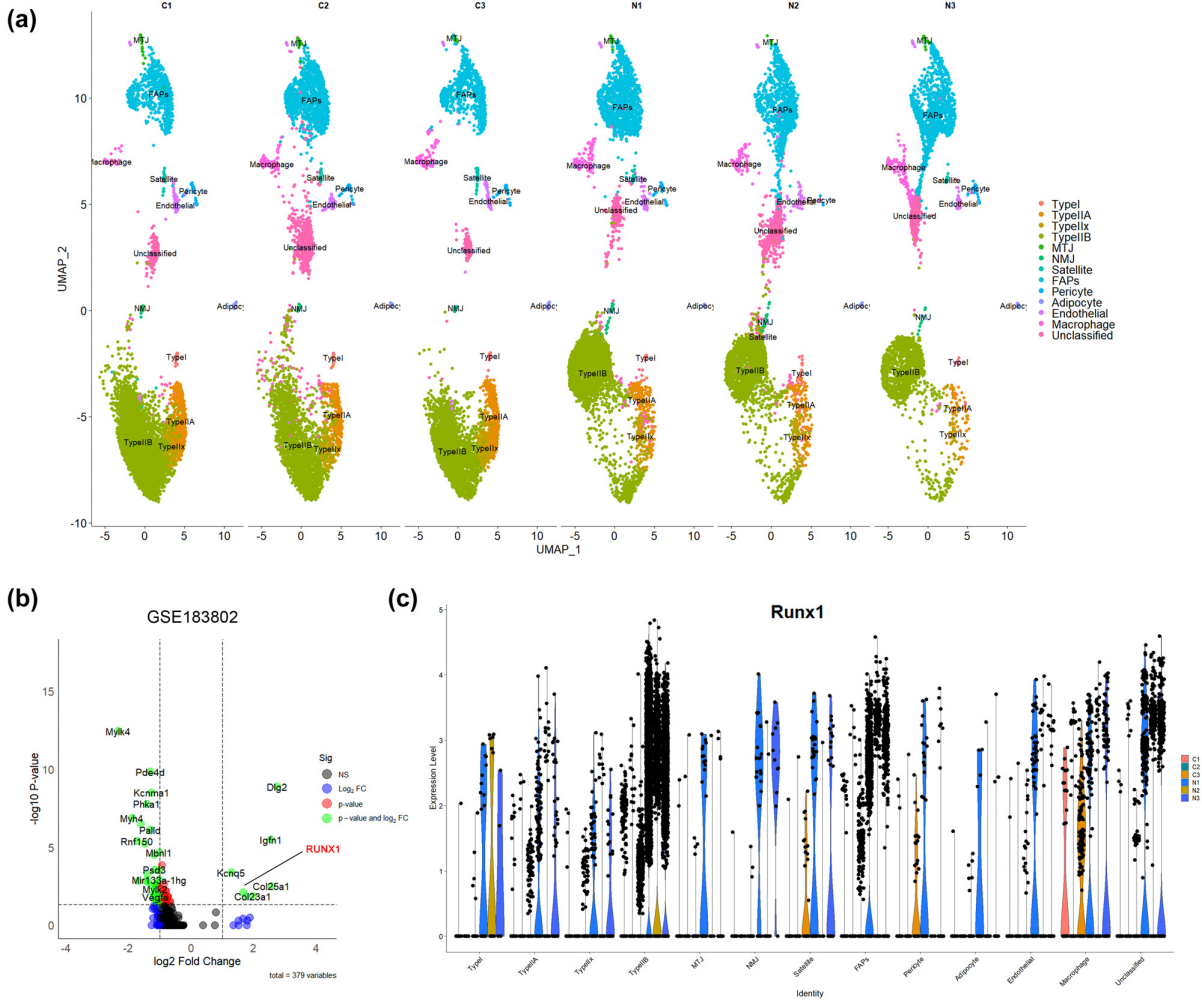
snRNA-seq. (a) Identification of 12 distinct cell populations from GSE183802 database via snRNA-seq. (b) Differential gene analysis reveals RUNX1 upregulation in denervated type I myonuclei. (c) RUNX1 expression significantly elevated in type I and type IIa myofibers clusters post-denervation.

A comprehensive review of previous studies revealed that the GSE183802 dataset provides snRNA-seq data from the GAS muscles of normal mice and mice subjected to denervation-induced muscle atrophy. This dataset retained 29,539 nuclei following quality control, comprising 15,739 nuclei from the normal group and 13,800 nuclei from the denervated group. Utilizing the Seurat package, the analysis identified 12 distinct cell populations, including type I myofibers, type II myofibers (IIa, IIx, and IIb), FAPs, MuSCs, NMJ cells, muscle-tendon junction cells, macrophages, endothelial cells, pericytes, adipocytes, and a group of unclassified nuclei (Figure 1a). To understand the molecular changes associated with denervation-induced muscle atrophy, differential gene expression analysis was conducted on type I myonuclei, comparing denervated and normal samples. This analysis revealed the significant upregulation of Dlg2, Col25a1, Igfn1, JUNB, Gadd45a, Runx1, and Kcng5, while genes such as Mylk4, Myh4, Rnf150, Rp1, Lrrfip1, Hs3st5, and Oxct1 were significantly downregulated (Figure 1b). Given its strong association with skeletal muscle atrophy, RUNX1 was selected for further investigation to determine its expression patterns across different cell populations. The findings demonstrated that RUNX1 expression was upregulated in type I, type IIa, and macrophage subclusters (Figure 1c), underscoring its potential role in mediating denervation-induced muscle atrophy.

### 3.2 Establishment of the sciatic nerve transection model for skeletal muscle atrophy

**Figure 2:**
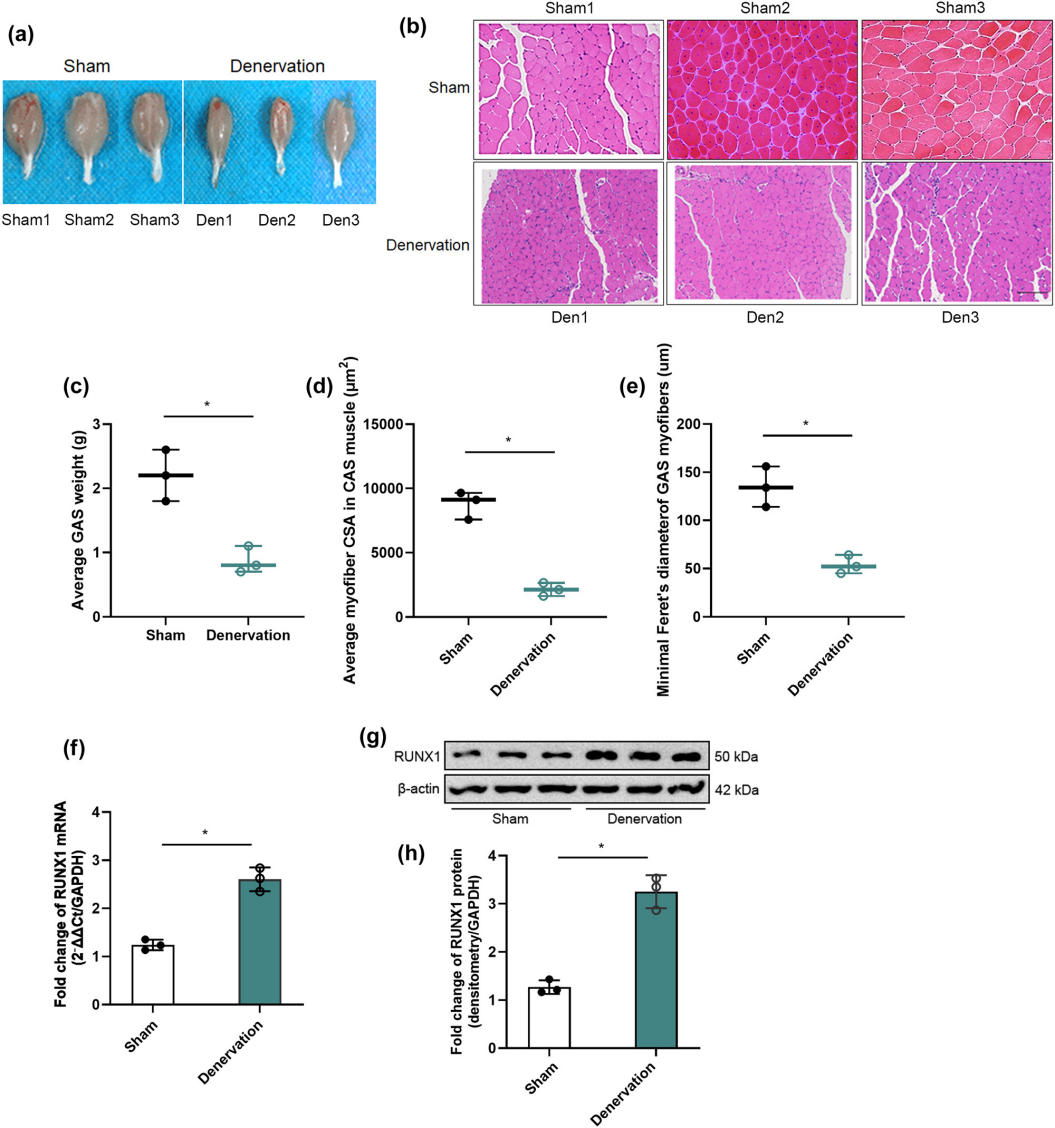
Sciatic nerve transection model. (a) Gross GAS morphology in sham vs denervation groups, 14 days post‐surgery. (b) HE‐stained GAS cross‐sections in sham vs denervation groups. (c) GAS wet weight in sham vs denervation groups. (d) Muscle fiber CSA in sham vs denervation groups. (e) Minimal Feret’s diameter in sham vs denervation groups. (f) RUNX1 mRNA levels in GAS by qRT-PCR in sham vs denervation groups. (g) RUNX1 protein levels in GAS by Western blot in sham vs denervation groups. (h) Densitometric quantification of RUNX1 protein levels normalized to GAPDH.

Sciatic nerve transection is a widely used experimental method for inducing skeletal muscle atrophy, providing a reliable model for investigating the mechanisms of muscle degeneration [1,10,11]. To assess the validity and consistency of this model, its effects were evaluated 14 days post-transection [12,13]. Figure 2a highlights the apparent atrophy of lower extremity muscles on the denervated side compared to the contralateral control side. Histological analysis of skeletal muscle atrophy is shown in Figure 2b, where HE staining of cross-sections demonstrates a significant reduction in GAS muscle fiber diameters in the injury group relative to the control group. This observation is further corroborated by a substantial decrease in the wet weight of GAS muscles, as illustrated in Figure 2c. Moreover, both the average (CSA, Figure 2d) and the minimal Feret’s diameter (Figure 2e) of GAS muscles were significantly reduced in the denervated group. Subsequent qRT-PCR analysis revealed that RUNX1 expression was significantly increased in the injury group compared to the control group, as evidenced by both mRNA levels (Figure 2f) and protein expression (Figure 2g and h).

### 3.3 RUNX1 overexpression in C2C12 cells enhances M1 polarization and muscle atrophy-related gene expression in macrophages

**Figure 3:**
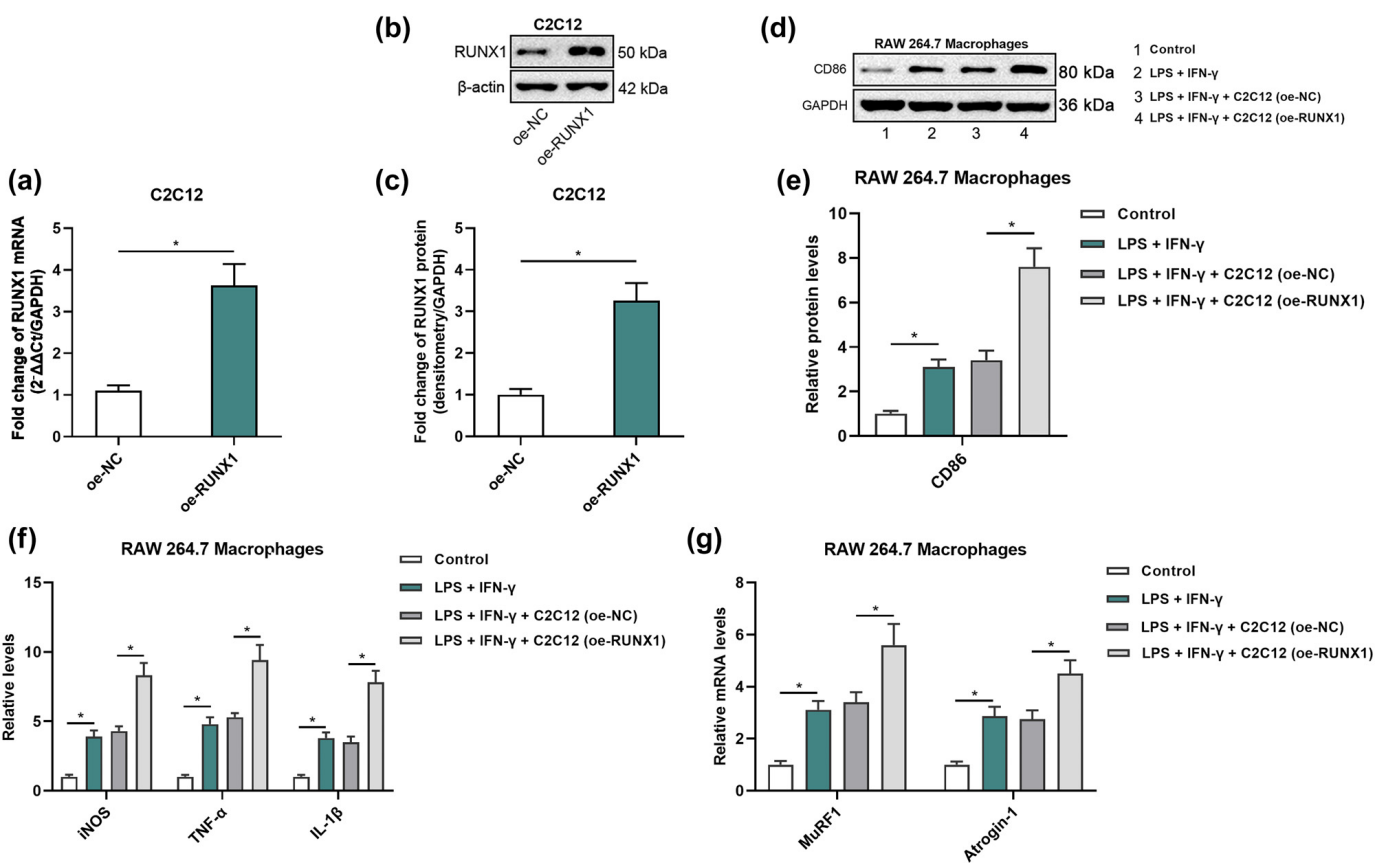
RUNX1 overexpression in C2C12 cells promotes M1 macrophage polarization. (a) qRT-PCR validation of RUNX1 mRNA overexpression in C2C12 cells (oe-NC vs oe-RUNX1). (b) Western blot of RUNX1 protein levels in C2C12 cells following transfection. (c) Densitometric quantification of RUNX1 protein normalized to GAPDH. (d) Western blot of CD86 in RAW264.7 macrophages treated with supernatant from oe-NC or oe-RUNX1 C2C12 cells. (e) Densitometric quantification of CD86 protein normalized to GAPDH. (f) ELISA measurement of iNOS, IL-1β, and TNF-α in macrophage culture supernatants. (g) qRT-PCR analysis of MuRF1 and Atrogin-1 mRNA levels in treated macrophages.

To confirm the efficiency of RUNX1 overexpression, C2C12 cells were transfected with either an empty vector (oe-NC) or a RUNX1 overexpression plasmid (oe-RUNX1) and cultured for 24 h. qRT-PCR and Western blot analyses confirmed a significant increase in RUNX1 mRNA and protein expression levels in the oe-RUNX1 group compared to the oe-NC group (Figure 3a–c). Supernatants from the transfected C2C12 cells were then collected and added to RAW 264.7 macrophages treated with LPS and IFN-γ to induce M1 polarization. Western blot analysis further demonstrated an upregulation of CD86 protein expression in the LPS + IFN-γ group (Figure 3d and e). ELISA results showed that the secretion levels of iNOS, IL-1β, and TNF-α were significantly elevated in the LPS + IFN-γ group compared to the untreated control group (Figure 3f). Additionally, qRT-PCR revealed a significant increase in MuRF1 and Atrogin-1 mRNA levels in macrophages treated with LPS and IFN-γ (Figure 3g), suggesting an association between M1 polarization and the expression of muscle atrophy-related genes. The addition of C2C12 supernatants from the oe-NC group did not significantly alter the expression of iNOS, IL-1β, TNF-α, CD86, MuRF1, or Atrogin-1 compared to the LPS + IFN-γ group. However, the presence of C2C12 supernatants from the oe-RUNX1 group further enhanced iNOS, IL-1β, and TNF-α levels (Figure 3f), CD86 protein expression (Figure 3e), and MuRF1 and Atrogin-1 mRNA levels (Figure 3g) in LPS + IFN-γ-treated macrophages compared to the LPS + IFN-γ + oe-NC group.

### 3.4 RUNX1 directly targets JUNB

**Figure 4:**
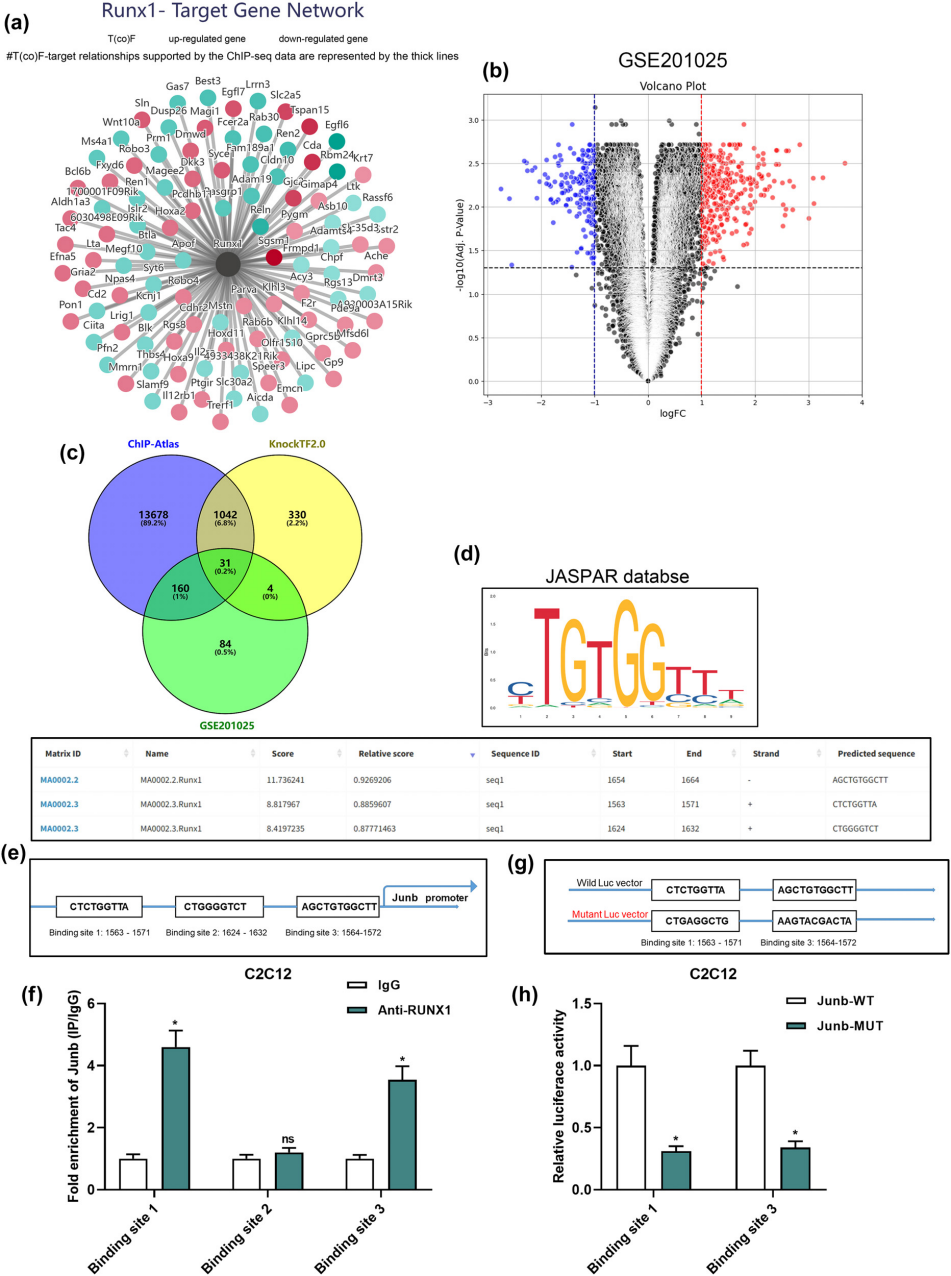
RUNX1 directly targets JUNB. (a) Identification of RUNX1 downstream targets using the KnockTF 2.0 database. (b) JUNB expression significantly upregulated in GSE102255 dataset. (c) Intersection analysis identified JUNB as a shared target gene of RUNX1. (d) Potential binding sites were predicted by JASPAR. (e) Schematic of the JUNB promoter region with three predicted RUNX1 binding motifs. (f) ChIP assays confirmed direct binding of RUNX1 to JUNB promoter regions (Sites 1 and 3). (g) Sequences of WT and MUT RUNX1 binding motifs in the JUNB promoter used for reporter constructs. (h) Dual-luciferase reporter assay comparing relative luciferase activity between the two groups.

To explore the regulatory mechanism of RUNX1, the KnockTF 2.0 database was utilized to identify its downstream target genes. This analysis revealed 1,425 negatively regulated genes and 756 positively regulated genes (Figure 4a). Complementing this, the ChIP-Atlas database predicted 14,910 potential downstream targets of RUNX1. By intersecting these predictions with genes upregulated in type I myonuclei identified from the GSE102255 dataset (Figure 4b), JUNB was identified as a shared target (Figure 4c). To validate that RUNX1 transcriptionally regulates JUNB, the JASPAR database was used to predict RUNX1 binding sites within the JUNB promoter region (Figure 4d). The analysis identified three putative binding sites. ChIP assays were then conducted to verify these predictions, demonstrating that RUNX1 specifically binds to Binding Sites 1 and 3, but not to Binding Site 2, on the JUNB promoter (Figure 4e and f). To further assess the functional importance of these binding sites, a dual-luciferase reporter assay was performed. The results revealed a significant reduction in luciferase activity in the JUNB-MUT group compared to the JUNB-WT group (Figure 4g and h), corroborating the regulatory influence of RUNX1 on JUNB transcription.

### 3.5 JUNB reverses RUNX1-induced M1 polarization

**Figure 5:**
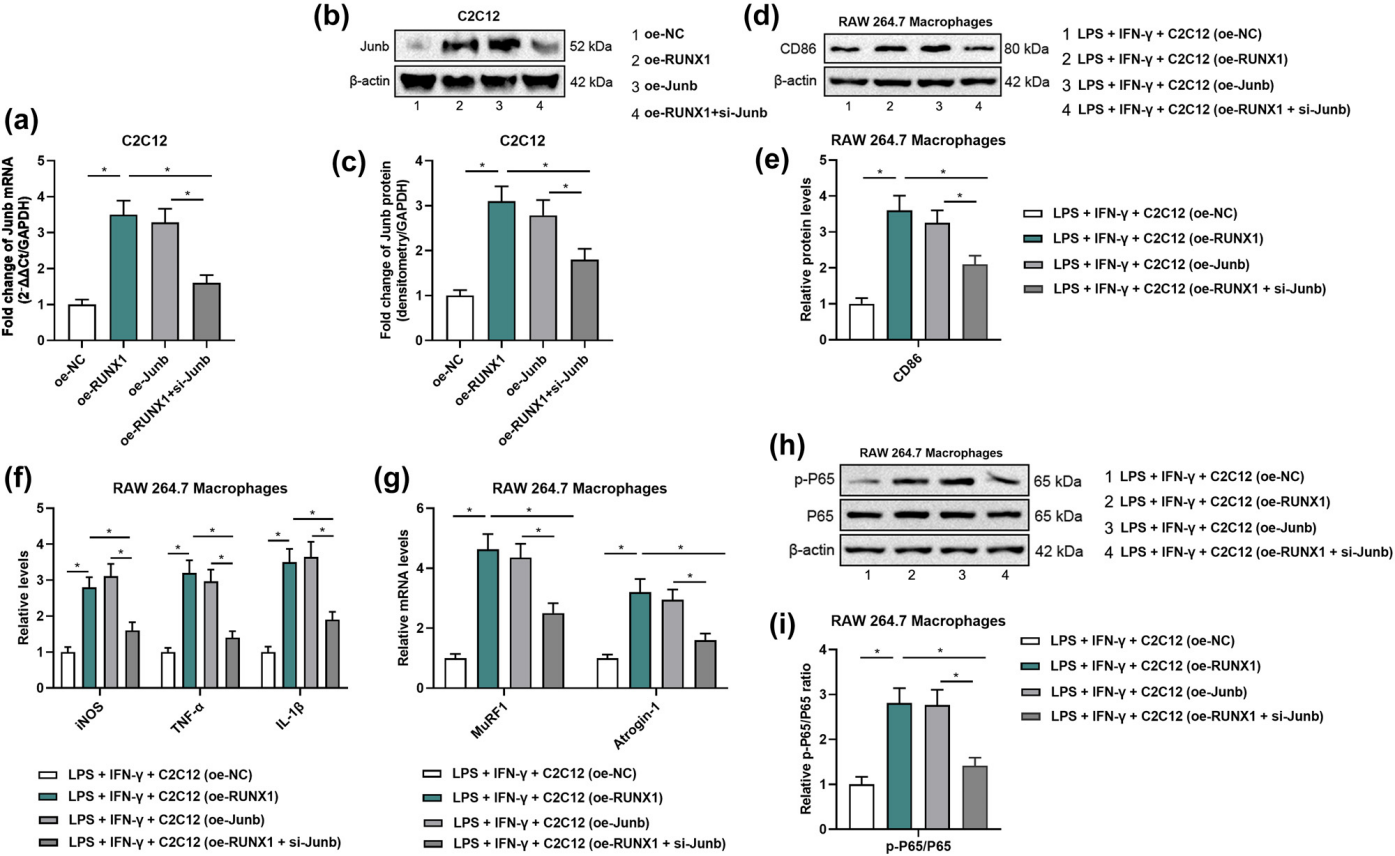
JUNB knockdown reverses RUNX1-induced M1 macrophage polarization via NF-κB. (a) qRT-PCR validation of JUNB knockdown efficiency in C2C12 cells transfected with si-JUNB vs control siRNA. (b) Western blot of JUNB protein levels in C2C12 cells after siRNA transfection. (c) Densitometric quantification of JUNB protein normalized to GAPDH. (d) Western blot of CD86 expression in RAW264.7 macrophages treated with conditioned medium from C2C12 cells (NC, oe-RUNX1, oe-RUNX1 + si-JUNB). (e) Densitometric quantification of CD86 normalized to GAPDH. (f) ELISA measurement of iNOS, IL-1β, and TNF-α in macrophage supernatants. (g) qRT-PCR analysis of MuRF1 and Atrogin-1 mRNA levels in treated macrophages. (h) Western blot of phosphorylated NF-κB p65 in macrophages exposed to conditioned media. (i) Densitometric quantification of p-p65 normalized to total p65.

C2C12 cells were transfected with either an empty vector (NC), a RUNX1 overexpression plasmid (oe-RUNX1), or a RUNX1 overexpression plasmid combined with JUNB siRNA (oe-RUNX1 + si-JUNB). qRT-PCR and Western blot confirmed successful transfection, with JUNB expression significantly reduced in the oe-RUNX1 + si-JUNB group compared with the oe-RUNX1 group (Figure 5a–c). Supernatants from the transfected C2C12 cells were subsequently added to RAW264.7 macrophages treated with LPS and IFN-γ to induce M1 polarization. RUNX1 overexpression resulted in significant increases in the levels of CD86 (Figure 5d and e), iNOS, IL-1β, TNF-α (Figure 5f), MuRF1, and Atrogin-1 (Figure 5g) compared with the NC group, whereas JUNB knockdown effectively reversed these effects. Moreover, analysis of the NF-κB pathway revealed that overexpression of RUNX1 significantly increased phosphorylated p65 levels, whereas knockdown of JUNB markedly reduced p-p65 expression (Figure 5h and i).

## 4 Discussion

RUNX1 has been widely studied in the context of denervation-induced muscle atrophy and NMJ remodeling, particularly in aging and stress-induced muscle dysfunction [14]. Previous research established RUNX1 as a critical transcription factor in the early stress response to denervation, with its upregulation linked to atrophy-related gene activation, including Fbxo32 (Atrogin-1) and Gadd45a [8,9]. In mild denervation models, such as nSod1KO mice, RUNX1 expression increases in response to NMJ instability, suggesting an adaptive role in maintaining NMJ integrity [14]. However, chronic denervation, aging, or sustained stress leads to persistent RUNX1 upregulation, which correlates with progressive NMJ dysfunction and sarcopenia. This dual role – protective in acute stress but deleterious in chronic settings – is further underscored by sex-specific differences and exercise interventions that reduce RUNX1 expression, ameliorating age-related muscle decline [7]. Despite these advances, RUNX1’s role in immune-mediated muscle atrophy, particularly through macrophage interactions, remained poorly defined. Macrophages are pivotal in muscle repair, balancing pro-inflammatory (M1) and anti-inflammatory (M2) polarization during injury and regeneration [9]. Persistent M1 polarization, marked by elevated IL-1β, TNF-α, and CD86, drives chronic inflammation and atrophy. Our findings align with this paradigm: RUNX1 overexpression in C2C12 myotubes promoted M1 polarization, amplifying pro-inflammatory cytokine secretion (e.g., IL-1β, TNF-α) and upregulating atrophy markers (MuRF1, Atrogin-1). This mirrors prior studies showing that M1 macrophages exacerbate proteolysis and inhibit regeneration [9]. Notably, RUNX1’s broader role in tissue remodeling is evident in fibrotic diseases like systemic sclerosis (SSc), where hypomethylation-driven RUNX1 overexpression accelerates ECM dysregulation [15]. These parallels reinforce RUNX1 as a molecular switch between adaptive stress responses and chronic degenerative pathways, positioning it as a therapeutic target to modulate inflammation and ECM dynamics in atrophy. The upstream mechanisms regulating RUNX1 expression following denervation remain unclear. Potential triggers may include oxidative stress reactive oxygen species (ROS), ischemia-reperfusion injury, or neurotrophic factors such as nerve growth factor and brain-derived neurotrophic factor. Future research will explore these possibilities using ROS inhibitors or receptor antagonists in both *in vitro* and *in vivo* models.

In this study, we identified JUNB as a novel downstream effector of RUNX1, mechanistically linking RUNX1 to NF-κB-driven inflammation and muscle degeneration. While JUNB-NF-κB interactions are well-documented in chronic inflammatory diseases [8], their role in denervation atrophy was unexplored. Our data reveal that RUNX1 directly binds the JUNB promoter, activating its transcriptional regulatory relationship conserved in fibrotic and muscular pathologies. For instance, in SSc, hypomethylation of both RUNX1 and JUNB drives ECM overproduction [15], paralleling our observation of JUNB upregulation in type I/IIa myonuclei post-denervation. This suggests that RUNX1-JUNB signaling is a shared axis in tissue remodeling across diverse conditions. Importantly, JUNB’s role in macrophage polarization extends beyond muscle atrophy. In endometriosis, JUNB collaborates with AP-1 members (e.g., FOS, FOSB) to drive early-stage M1 polarization and inflammation [16], mirroring our findings in denervated muscle. Conversely, late-stage endometriosis shifts toward M2 polarization, emphasizing JUNB’s context-dependent role in immune modulation. Our work extends these insights by demonstrating that RUNX1-induced JUNB amplifies NF-κB signaling (via p65 phosphorylation), creating a feedforward loop that sustains M1 polarization and cytokine release (e.g., IL-1β, TNF-α). This mechanism aligns with studies in inflammatory skin diseases, where JUNB deficiency enhances NF-κB activation by derepressing SQSTM1/p62, exacerbating inflammation [17]. Similarly, in ischemic stroke, JUNB/NF-κB modulation by Sparganin C promotes microglial M2 polarization, attenuating injury [17]. These parallels underscore JUNB’s universal role as a rheostat for NF-κB activity and macrophage phenotypes.

Critically, our study bridges RUNX1 to the JUNB-NF-κB axis, revealing its dual impact on immune and muscle cells. RUNX1 overexpression in myotubes not only upregulated JUNB but also induced a paracrine inflammatory cascade that polarized macrophages toward M1, further amplifying muscle atrophy markers. This crosstalk highlights the microenvironmental interplay between myocytes and macrophages in driving atrophy. The reversal of these effects by JUNB knockdown confirms its centrality in RUNX1-mediated pathology. Notably, myeloid-specific JUNB deletion in cerebral malaria models reduces IL-1β/TNF production and shifts macrophages toward a regulated phenotype, improving survival [18], a finding that dovetails with our observation that JUNB silencing suppresses M1 polarization and NF-κB activation. Collectively, these data position JUNB as a linchpin connecting transcriptional regulation (via RUNX1) to inflammatory signaling (via NF-κB) in muscle atrophy. A limitation of our study is the use of the immortalized RAW 264.7 macrophage cell line, which may amplify RUNX1-induced signaling and does not fully replicate the complex interactions between macrophages, satellite cells, and fibroblasts in muscle. Future studies should isolate primary macrophages from denervated muscles and employ co-culture systems to validate our findings and further elucidate the physiological relevance.

## 5 Conclusion

Our findings establish the RUNX1-JUNB-NF-κB axis as a central driver of denervation-induced muscle atrophy. RUNX1 activates JUNB transcription, which in turn amplifies NF-κB signaling to promote M1 macrophage polarization, pro-inflammatory cytokine release, and atrophy-related gene expression. JUNB knockdown reverses these effects, validating its role as a critical mediator. Given that our experiments were conducted in a mouse model, further studies using human muscle tissues or primary human myotubes will be necessary to assess clinical relevance. Future directions include (1) validating RUNX1–JUNB–NF-κB signaling in patient-derived cells, (2) exploring upstream triggers of RUNX1 activation post-denervation, and (3) investigating additional downstream targets of RUNX1 to map the broader regulatory network.

## Supplementary Material

Supplementary Figure
