# Supplementary material for: RUNX1 promotes denervation-induced muscle atrophy by activating the JUNB/NF-κB pathway and driving M1 macrophage polarization
Source: Open Life Sci. 2025 Aug 18;20(1):20251157. doi: 10.1515/biol-2025-1157 (PMC12412371; doi:10.1515/biol-2025-1157)
Supplement: Supplementary Figure [file biol-2025-1157-sm.pdf]

Supplementary material

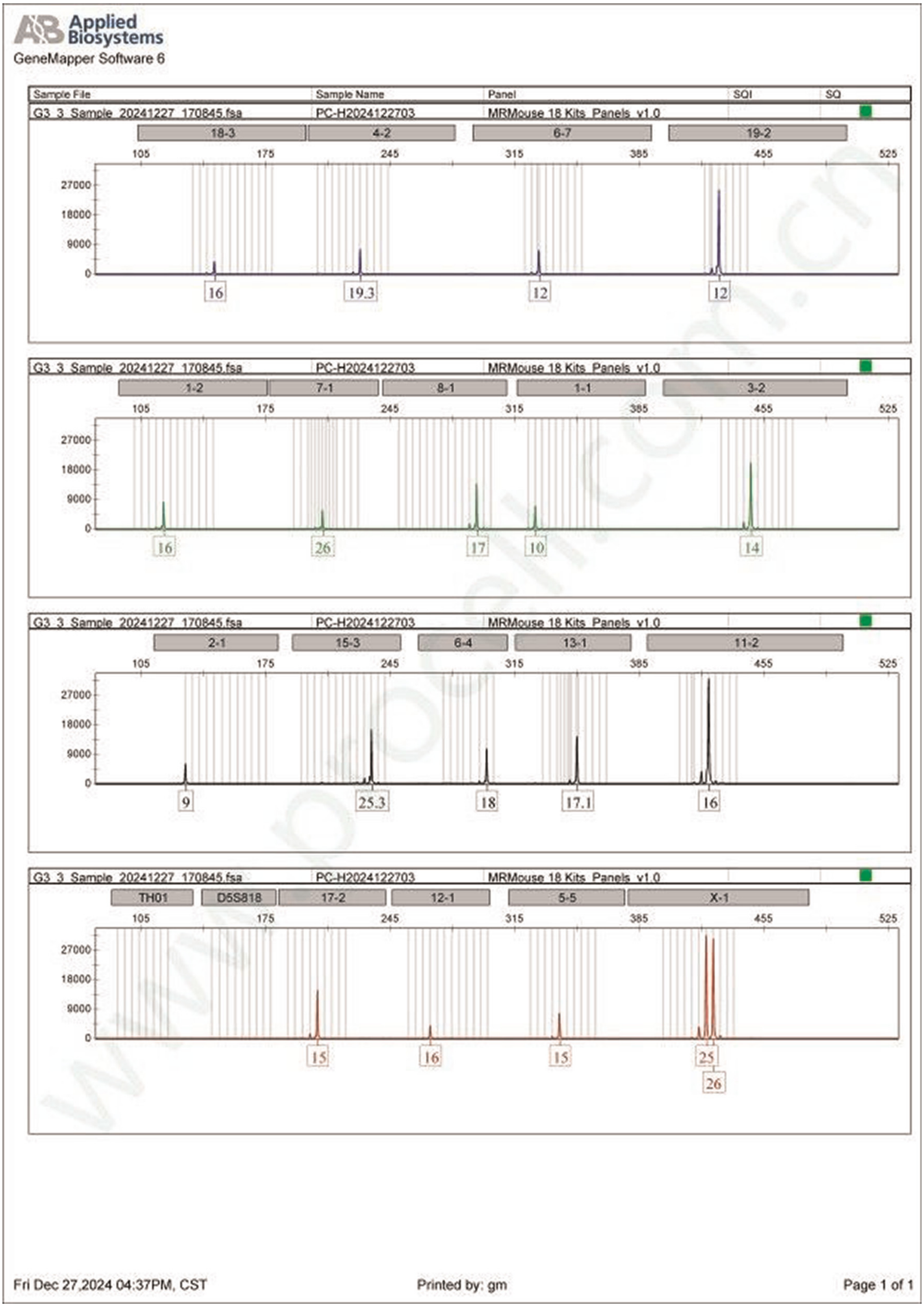

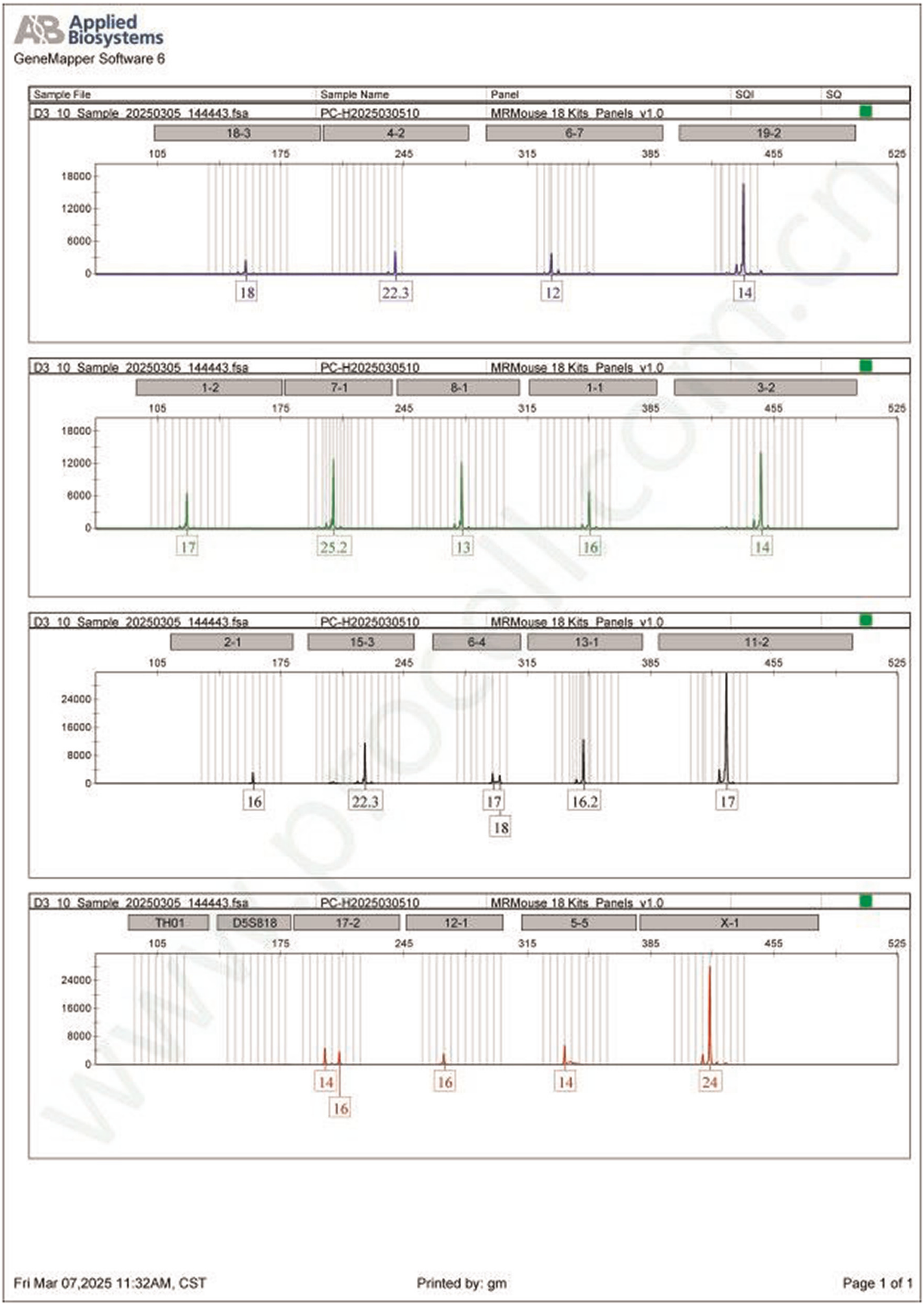
Applied Biosystems
GeneMapper Software 6
Sample File
Sample Name
Panel
SQI
SQ
D3_10_Sample_20250305_144443.fsa
PC-H2025030510
MRMouse 18 Kits_Panels_v1.0
18-3
4-2
6-7
19-2
18
22.3
12
14
1-2
7-1
8-1
1-1
3-2
17
25.2
13
16
14
2-1
15-3
6-4
13-1
11-2
16
22.3
17
18
16.2
17
TH01
D5S818
17-2
12-1
5-5
X-1
14
16
16
14
24
Fri Mar 07,2025 11:32AM, CST
Printed by: gm
Page 1 of 1

RAW 264.7 STR site information

18-3	18	15-3	22.3
6-7	12	12-1	16
5-5	14	6-4	17,18
X-1	24	4-2	22.3
1-2	17	3-2	14
7-1	25.2	2-1	16
8-1	13	13-1	16.2
1-1	16	11-2	17
19-2	14	17-2	14,16

C2C12 STR site information

18-3	16	15-3	25.3
6-7	12	12-1	16
5-5	15	6-4	18
X-1	25,26	4-2	19.3
1-2	16	3-2	14
7-1	26	2-1	9
8-1	17	13-1	17.1
1-1	10	11-2	16
19-2	12	17-2	15

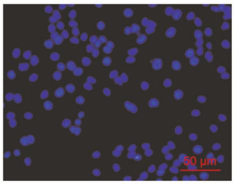

Hoechst staining of RAW 264.7 cells

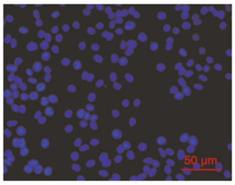

Hoechst staining of C2C12 cells

Figure S1. STR profiling.
